# Circular RNA *cir-ITCH* Promotes Osteosarcoma Migration and Invasion through *cir-ITCH*/miR-7/EGFR Pathway

**DOI:** 10.1177/1533033819898728

**Published:** 2020-01-21

**Authors:** Hongbo Li, Min Lan, Xingen Liao, Zhiming Tang, Chunli Yang

**Affiliations:** 1 Department of Orthopedics, Jiangxi Provincial People’s Hospital Affiliated to Nanchang University, Nanchang, China

**Keywords:** cir-ITCH, miR-7, EGFR, migration, invasion, osteosarcoma

## Abstract

Recent studies have suggested that circular RNAs play an important role in the progression of various cancers. We aimed to investigate the possible role of *cir-ITCH* in osteosarcoma. In this study, we performed experiments with the human osteoblast cell line hFOB1.19 and several osteosarcoma cancer cell lines and the results showed that the expression of *cir-ITCH* in osteosarcoma cancer cell lines was significantly upregulated compared to that in the human osteoblast cell line. In addition, the results showed that *cir-ITCH* could promote the migration, invasion, and growth of osteosarcoma cells. Further mechanistic studies revealed that *cir-ITCH* could enhance epidermal growth factor receptor (EGFR) expression by reducing the level of miR-7. Increased EGFR phosphorylation was found to be concomitant with high expression of EGFR. We determined that *cir-ITCH*-mediated increase in the migration and invasion of osteosarcoma cells was dependent on EGFR phosphorylation. In conclusion, our research uncovered an important role of the *cir-ITCH*/miR-7/EGFR pathway in the migration and invasion of osteosarcoma cells and suggested that *cir-ITCH* may be a prognostic marker and a promising therapeutic target for osteosarcoma.

## Introduction

Osteosarcoma (OS) is the most common bone cancer and the third most frequent malignancy of adolescents and is characterized by poor survival.^1^ Moreover, in the past several decades, the treatment of OS has not changed. The main treatments are still surgery and nonspecific chemotherapy.^2,3^ Tumor cells can invade and migrate to other tissues, such as the brain and prostate, which is the main cause of death.^4^ The 5-year survival of patients with OS is only 10%, and metastasis is responsible for most deaths.^5^ The precise mechanisms of OS metastasis remain unclear and require further study. Understanding the mechanism would provide a theoretical basis for developing targeted therapy that could be designed to specifically inhibit the metastasis of OS.

An increasing number of studies have elucidated that noncoding RNAs (ncRNAs) play an important role in the progression of cancer.^6^ Circular RNAs (circRNAs) are a novel class of widely expressed and diverse RNAs that can regulate mammalian gene expression.^7^ CircRNAs are 100 bp to 4 kb in size and covalently closed loops with linked 5′ and 3′ ends, which helps to resist digestion by RNase.^8,9^ The main function of circRNA is acting as a microRNA (miRNA) sponge and regulating the miRNA target genes through miRNA repression.^10^

Increasing evidence has shown that circRNAs play important roles in various cancer cellular activities, such as cell cycle progression, proliferation, and metastasis. Through the database analysis of circular RNA reported by Memczak *et al*,^11^ we found that *cir-ITCH* spans several E3 ubiquitin (Ub) exons.^9,12^ The reports indicated that *cir-ITCH* has binding sites in many miRNAs, such as miR-214, miR-17, miR-7, miR-216b, and miR-128, suggesting that it may act as a miRNA sponge.^10^ It has been found that *cir-ITCH* plays an inhibitory role in both oesophageal squamous cell carcinoma and colorectal cancer and also suppresses lung cancer proliferation.^12,13,14^

In our research, we found that *cir-ITCH* was an oncogene that was upregulated in OS. Furthermore, *cir-ITCH* could decrease miR-7 expression levels, thereby leading to activation of the epidermal growth factor receptor (EGFR) pathway accompanied by high metastasis ability. This study revealed a critical role of *cir-ITCH* in OS progression and new mechanisms leading to OS invasion and metastasis.

## Materials and Methods

### Cell Culture

SJSA-1 and U2OS cells were obtained from Cell Bank, Type Culture Collection, Chinese Academy of Sciences (Shanghai, China). SJSA-1 cells were cultured with Dulbecco’s modified Eagle’s medium (DMEM) supplemented with glucose and 10% fetal bovine serum (FBS). U2OS cells were grown in McCoy 5A medium with 10% FBS. All cells were cultured in cell incubators with 5% CO_2_ at 37°C.

### Plasmid Construction and Transfection

The sequence of *cir-ITCH* was cloned by polymerase chain reaction (PCR) and inserted into the pcDNA3.1 vector. All small interfering RNAs were obtained from RiboBio (Guangdong, China). The indicated cells were transiently transfected with 0.1 µmol/l mimics of miR-7 or control (Bioneer, Daejeon, Korea) with Lipofectamine 2000.

### RNA Extraction and qRT-PCR Analysis

RNA was isolated using a Roche kit (Roche Applied Science, Basel, Switzerland) (TriPure Isolation Reagent). Complementary DNA (cDNA) was synthesized using a cDNA synthesis kit. Quantitative real-time polymerase chain reactions (qRT-PCRs) were carried out with a SYBR Green Kit (ABI, Warrington, United Kingdom). Glyceraldehyde 3-phosphate dehydrogenase was used as the endogenous reference gene. The results were confirmed by 3 independent experiments. The primer sequences have been published previously.^1^

**Table:** 

Gene	Forward (5′-3′)	Reverse (5′-3′)
*cir-ITCH*	GCAGAGGCCAACACTGGAA	TCCTTGAAGCTGACTACGCTGAG
*Linear ITCH*	TAGACCAGAACCTCTACCTCCTG	TTAAACTGCTGCATTGCTCCTTG
*GAPDH*	CCATGACCCCTTCATTGACC	TTGATTTTGGAGGGATCTCG
*MiR-7*	TGGAAGACTAGTGATTTTGTTT	AGACTGTGATTTGTTGTCGATT

### Cell Growth Assay

Cell Counting Kit-8 (CCK-8; Dojindo, Kumamoto, Japan) was used to identify the cell growth rate. A total of 3000 cells were plated onto a 96-well plate. At day 0, day 1, day 2, day 3, and day 4, 10-µL CCK-8 was added to the medium, and the optical density at 450 nm was tested 2 hours later.

### Wound-Healing Assay

Cells were placed onto 6-well cell culture dishes and cultured for 24 hours to achieve 100% confluence. Scratches were made by 200-µL pipette tips across the cell layers. The cells were washed with 10-mL phosphate-buffered saline solution 3 times and then incubated in serum-free media for 24 hours. At 0 and 24 hours, images were taken, and the gap length was calculated.

### Cell Invasion Assays

Transwell assays with Matrigel were used to measure cancer cell invasion with different treatments. A total of 1 × 10^5^ cancer cells were placed in the upper chamber with DMEM without FBS. Then, 700 µL of complete DMEM was added to the lower chamber. The noninvasive cells on the upper side of the membrane were removed after 48 hours. Subsequently, the membranes were fixed with 4% paraformaldehyde and 0.1% crystal violet.

### Western Blot

Western blot analysis of lysed OS cells was performed as previously described.^2^ Anti-EGFR, anti-phospho-EGFR 1068, anti-Erk1/2, and anti-phospho-Erk1/2 antibodies were purchased from Cell Signaling Technology (Danvers, MA, USA), and anti-β-actin antibody was obtained from Merck Millipore Billerica.

### Statistical Analyses

The results were analyzed by SPSS (Advanced statistical procedures companion, New Jersey, Prentice Hall Inc.Norusis) and GraphPad Prism (GraphPad Software, La Jolla, CA, USA). All the results are shown as the mean ± standard deviation. Differences between groups were calculated by Student *t* test.^3^ *P* < .05 was statistically significant.

## Results

### Cir-ITCHIs Highly Expressed in OS

**Figure 1.**
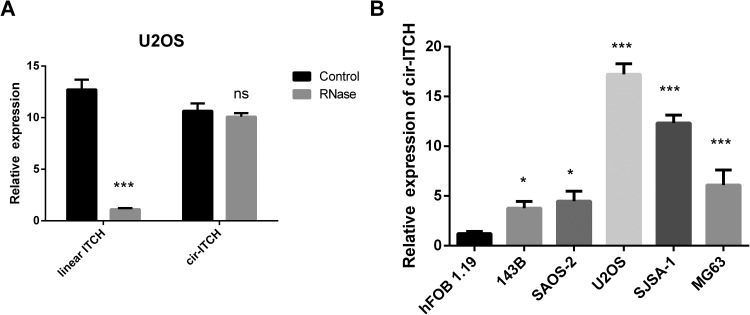
The expression of *cir-ITCH* in osteosarcoma (OS). A, quantitative real-time polymerase chain reaction (qRT-PCR) was used to identify linear *ITCH* and *cir-ITCH* expression in the OS cancer cell line U2OS. B, qRT-PCR revealed the expression of *cir-ITCH* in different OS cell lines. Data are shown as the mean ± standard deviation (n = 3).

The existence and important functions of *cir-ITCH* in several cancers have been reported,^12^ and we speculated that *cir-ITCH* may contribute to the progression of OS. As there are no previous reports on the expression of *cir-ITCH* in OS, we carried out PCR to identify whether *cir-ITCH* was expressed in OS. A special characteristic of circRNAs is that they are resistant to degradation by RNase, which can degrade linear RNAs in a 3′-5′ direction. The results showed that the linear *ITCH* messenger RNA was degraded by RNase, while *cir-ITCH* was resistant to it in the U2OS cell line (Figure 1A). This result confirmed the expression of *cir-ITCH* in the OS cell line. We also identified the expression of *cir-ITCH* in other OS cell lines by qRT-PCR. Compared to that in the human osteoblast hFOB 1.19 cell line, the expression of *cir-ITCH* was higher in OS cells (Figure 1B). In summary, we confirmed the presence of *cir-ITCH* in OS and found that *cir-ITCH* expression was higher in tumors than in normal cells.

### Cir-ITCH Promotes the Growth of OS Cells

**Figure 2.**
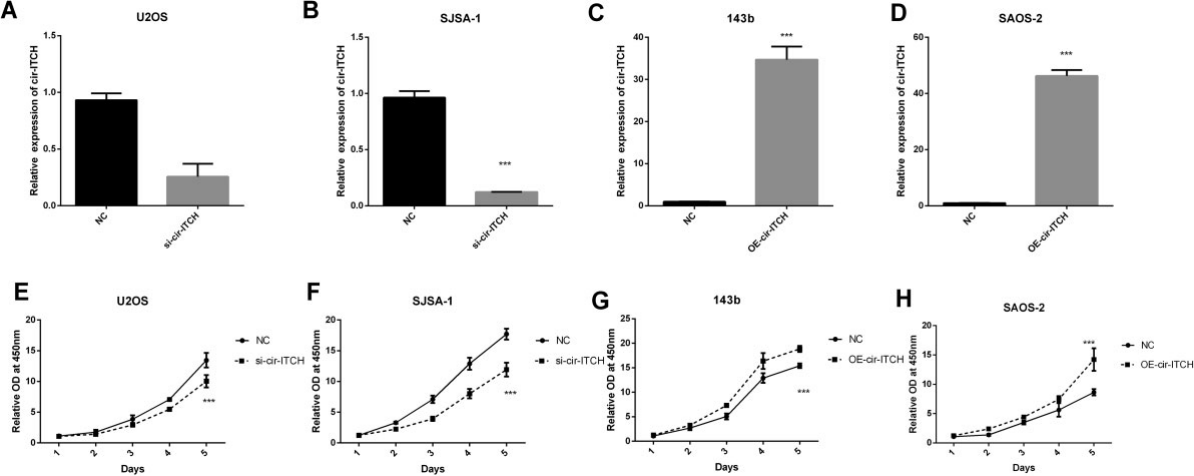
*cir-ITCH* promotes OS cell growth. A-B, quantitative real-time polymerase chain reaction (qRT-PCR) for *cir-ITCH* in U2OS (A) and SJSA-1 (B) cells treated with *cir-ITCH* or nonsense small interfering RNAs as described in the “Materials and Methods” section. C-D, qRT-PCR for *cir-ITCH* in143b (C) and SAOS-2 cells (D), which were transfected with *cir-ITCH* vector or empty vector. E-F, Cell Counting Kit-8 (CCK-8) was used to determine the effect of *cir-ITCH* silencing on U2OS (E) and SJSA-1 cells (F). G-H, CCK-8 was used to detect the effect of *cir-ITCH* overexpression on 143b (G) and SAOS-2 cells (H). Data are shown as the mean ± standard deviation (n = 3, *P < 0.05, **P < 0.01, ***P < 0.001 compared with the control).

To investigate the roles of *cir-ITCH* in OS, we carried out RNA interference to knock down *cir-ITCH* expression in U2OS and SJSA-1 cells (Figure 2A and B) and transfected a *cir-ITCH* overexpression plasmid into the 143b and SAOS-2 cell lines (Figure 2C and D). Cell Counting Kit-8 was used to identify the effect of *cir-ITCH* on OS cell growth. Silencing *cir-ITCH* impaired the proliferation of U2OS and SJSA-1 cells (Figure 2E and F), whereas overexpression of *cir-ITCH* promoted 143b and SAOS-2 cell growth (Figure 2G and H). We indicated that *cir-ITCH* could affect the growth rate of tumors by both overexpression and silencing of *cir-ITCH*.

### Cir-ITCH Induces the Migration and Invasion of OS Cancer Cells

**Figure 3.**
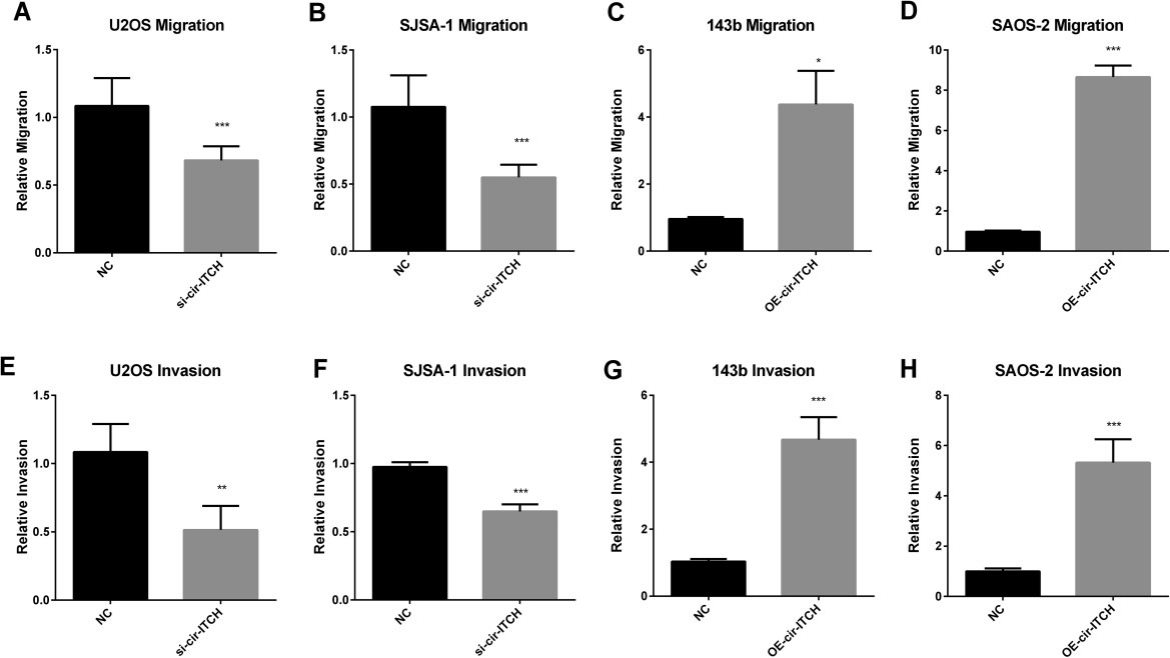
*cir-ITCH* promotes osteosarcoma (OS) migration and invasion. A-D, Migration was detected by wound healing assay in cells transfected with the indicated plasmids. E-F, Invasion assays were performed in OS cells transfected with the indicated plasmids. The results are shown as the mean ± standard deviation (n = 3, *P < 0.05, **P < 0.01, ***P < 0.001 compared with the control).

As metastasis is the main cause of death in patients with OS, we wanted to investigate whether *cir-ITCH* could influence the metastasis of OS. Due to the importance of migration and invasion in metastasis, we assessed the influence of *cir-ITCH* on OS migration by conducting a wound healing assay and identified invasion with a Transwell assay. The results showed that silencing *cir-ITCH* attenuated the migration and invasion of U2OS (Figure 3A and B) and SJSA-1 cells (Figure 3E and F), whereas overexpression of *cir-ITCH* dramatically promoted the migration and invasion of OS cells. Thus, the data above showed that *cir-ITCH* could promote the metastasis ability of OS.

### Cir-ITCH Decreases the Expression of miR-7

**Figure 4.**
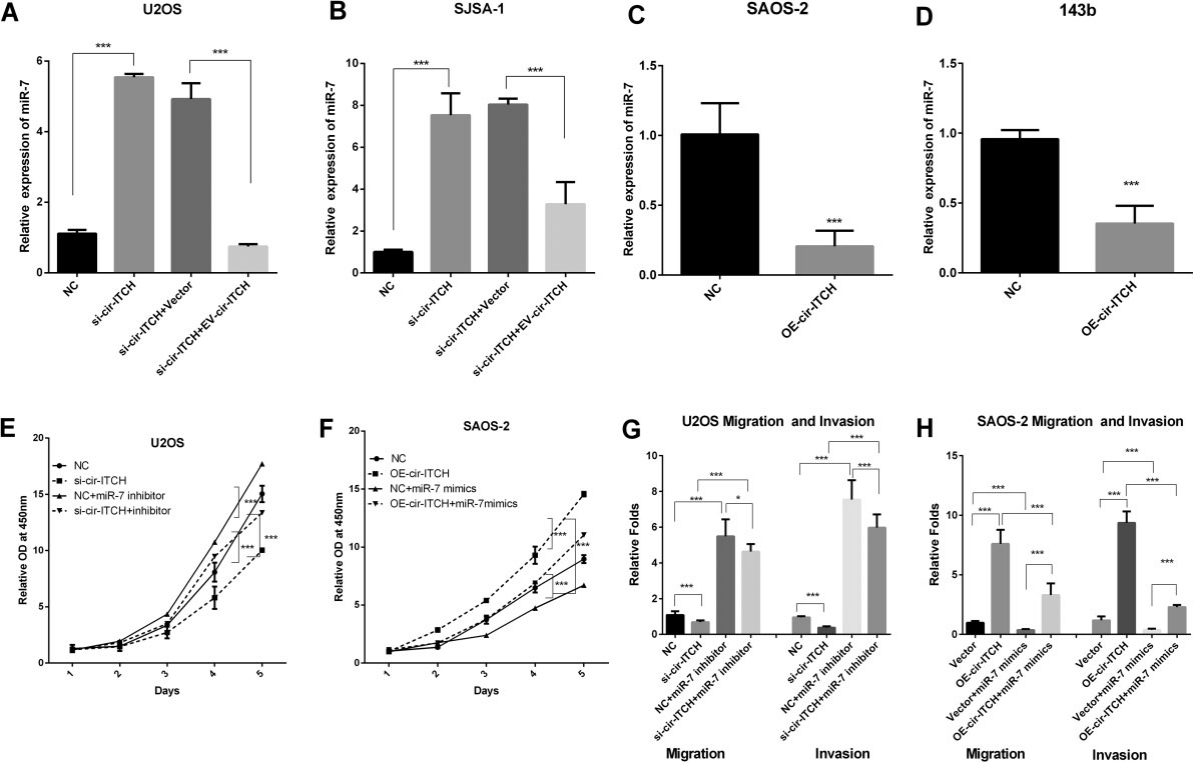
*cir-ITCH* affects OS progression through miR-7. A-B, The relative levels of miR-7 were analyzed by qRT-PCR in U2OS2 (A) and SJSA-1 (B) cells. Rescue experiments were carried out with transfections of vector or *cir-ITCH* vector in si-cir ITCH cells and then miR-7 expression were detected by qRT-PCR. C-D, The relative levels of miR-7 were analyzed by qRT-PCR in SAOS2 (C) and 143b (D)cells, which were transfected with NC and *cir-ITCH* vector. E, Cell Counting Kit-8 was carried out in U2OS NC and U2OS si-*cir-ITCH* cells that were transfected with miR-7 inhibitor. F, CCK-8 was used to detect cell growth in SAOS-2 NC and OE-*cir-ITCH* cells transfected with NC or miR-7 mimics. G-H, Wound healing and invasion assays were carried out in U2OS (G) and SAOS-2 (H) cells with the indicated treatments.

Next, we investigated the potential mechanism by which *cir-ITCH* influences migration and invasion in our study. We speculated that *cir-ITCH* could regulate the expression of a miRNA, as many previous reports have shown.^6,11^ We used miRanda and TargetScan software to predict *cir-ITCH*-binding miRNAs. The results showed that miR-17, miR-7, miR-128, miR-216b, and miR-214 may be potential target miRNAs of *cir-ITCH*. Then, we carried out qRT-PCR to investigate the influence of *cir-ITCH* on the miRNAs. The results showed that silencing *cir-ITCH* increased the expression of miR-7 in U2OS and SJSA-1 cells (Figure 4A and B), and re-expression of cir-ITCH blocked the expression of miR-7. In addition, overexpression of *cir-ITCH* decreased the expression of miR-7 (Figure 4C and D). Silencing of *cir-ITCH* could inhibit the growth, migration, and invasion of U2OS cells, while the inhibitor of miR-7 could block these effects on U2OS cells (Figure 4E and G). The function of miR-7 was also identified in SAOS-2 cells (Figure 4F and H). These findings indicate that *cir-ITCH* promotes OS metastasis and cell growth by inhibiting the level of miR-7.

### Cir-ITCH Activates the EGFR/ERK Signaling Pathway via miR-7

**Figure 5.**
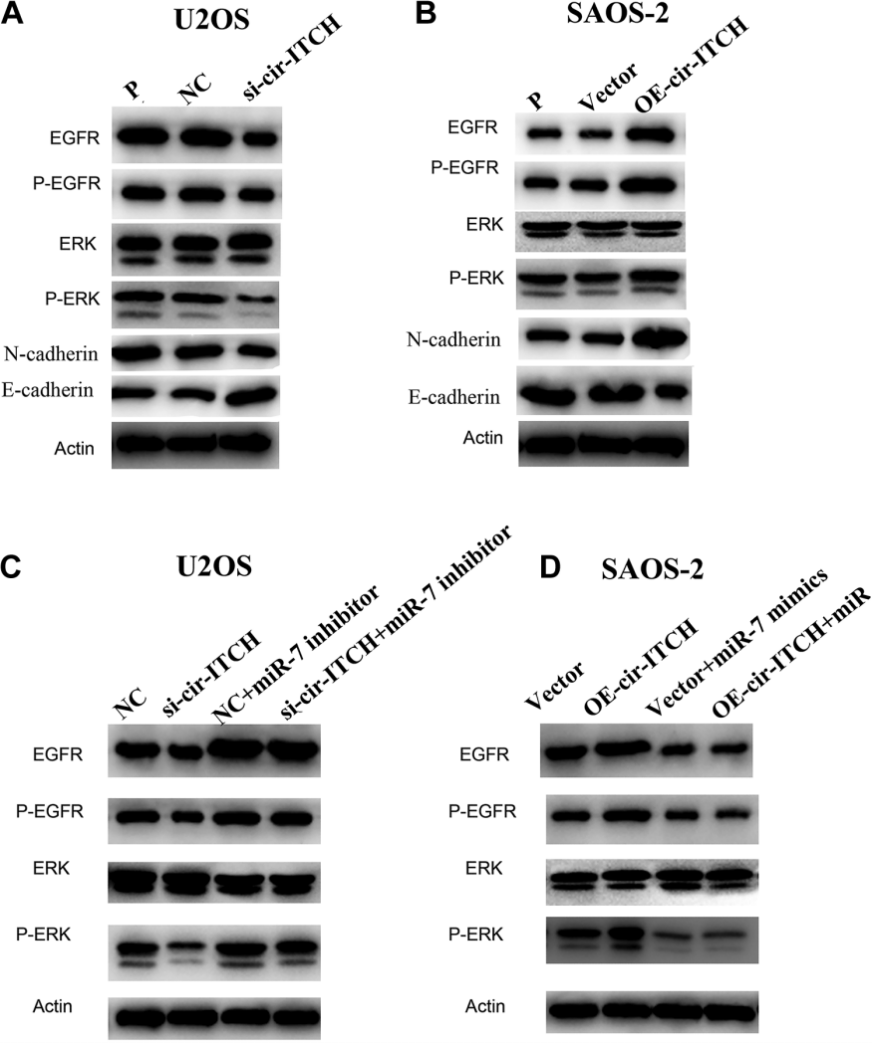
*cir-ITCH* increases EGFR protein levels and activates the EGFR/ERK pathway. A, Western blot was performed to identify the expression of EGFR/p-EGFR, ERK/p-ERK, and EMT markers in U2OS NC and si-*cir-ITCH* cells. B, Western blot was performed to identify the expression of EGFR/p-EGFR, ERK/p-ERK, and EMT markers in SAOS-2 cells transfected with control vector or *cir-ITCH* vector. C, The relative levels of the indicated proteins were analyzed by Western blot in U2OS2 NC and si-*cir-ITCH* cells treated with miR-7 inhibitor. D, The relative levels of the indicated proteins were analyzed by Western blot in SAOS-2 NC and OE-*cir-ITCH* cells transfected with NC and miR-7 mimics.

As previous studies have shown that miR-7 could regulate the progression of several cancers through the EGFR pathway,^15-18^ we carried out Western blotting to confirm whether *cir-ITCH* could activate the EGFR pathway via miR-7, thereby increasing the growth and metastasis of OS cells. In U2OS cells, the level of total and phosphorylation EGFR were downregulated with *cir-ITCH* silencing. Silence of *cir-ITCH* also leaded to the upregulation of E-cadherin and downregulation of N-cadherin (Figure 5A). The effects of *cir-ITCH* silencing were reversed by miR-7 inhibitors partially by EGFR/extracellular regulated protein kinases (ERK) activation (Figure 5C). On the other hand, overexpression of *cir-ITCH* increased EGFR protein level and activated EGFR/ERK pathway companied with E-cadherin upregulation and N-cadherin downregulation (Figure 5B), whereas the co-transfection of miR-7 mimics significantly reversed these effects in SAOS-2 cells (Figure 5D). These results showed a positive regulatory relationship between *cir-ITCH* and EGFR pathway activation.

### The *Cir-ITCH*/miR-7/EGFR Axis Is Important for the Migration and Invasion of Osteosarcoma Cancer Cells

**Figure 6.**
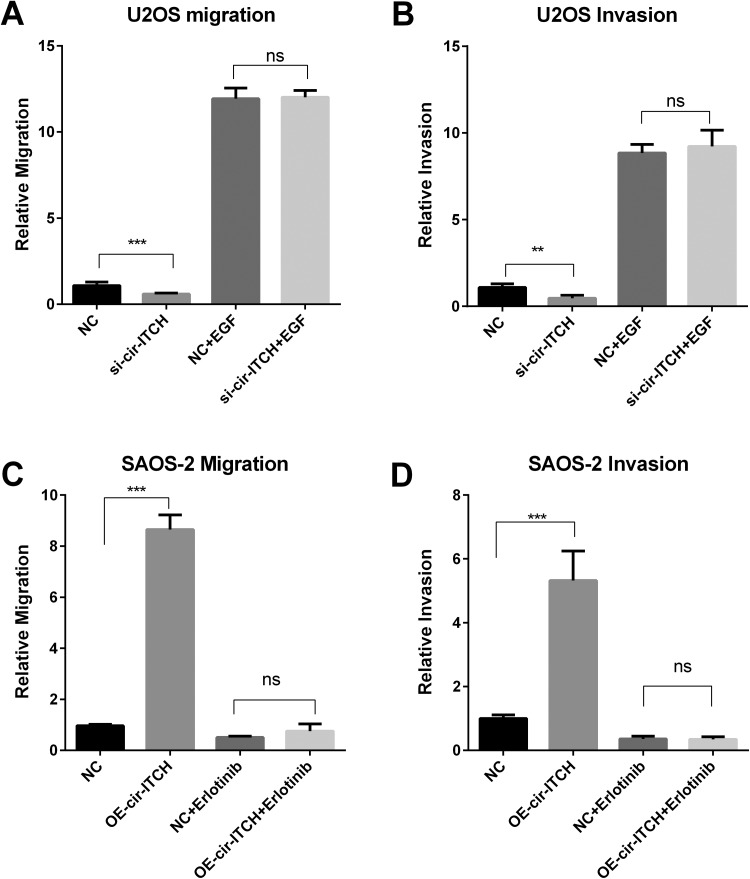
EGFR pathway activation promotes the migration and invasion of osteosarcoma cells. A-B, Wound healing assays and invasion assays were performed to identify the metastasis of U2OS cells treated with or without EGF (10 ng/mL). C-D, Migration and invasion assays were carried out to detect the metastasis of SAOS-2 cells treated with or without erlotinib (10 ng/mL).

As the EGFR pathway plays important roles in metastasis of cancer,^19^ we confirmed the role of the EGFR pathway in *cir-ITCH*-induced metastasis by the addition of EGFR activator and inhibitor. As an activator of EGFR, epithelial growth factor (EGF) could block the reduction in metastasis induced by *cir-ITCH* silencing (Figure 6A and B). Conversely, erlotinib, an inhibitor of EGFR phosphorylation, blocked the *cir-ITCH* overexpression-induced metastasis of SAOS-2 cells (Figure 6C and D). These findings indicate that *cir-ITCH* activates the EGFR/ERK signaling pathway, leading to OS metastasis.

## Discussion

Previously, *cir-ITCH* has been identified to repress the progression of oesophageal squamous cell carcinoma and colorectal cancer by the Wnt/β-catenin pathway.^20,12^ However, the function of *cir-ITCH* has not been elucidated in OS. In our research, we employed the human osteoblast cell line hFOB1.19 and several OS cancer cell lines. The results showed that the expression of *cir-ITCH* in OS cancer cell lines was upregulated significantly compared to that in the human osteoblast cell line as demonstrated by TaqMan-based qRT-PCR (Figure 1). These results suggest an important role for *cir-ITCH* in OS cancer.

In our study, CCK-8 assays showed that *cir-ITCH* mediated the promotion of cell growth. Moreover, the migration and invasion assay showed that *cir-ITCH* could promote the metastasis of OS cancer cells (Figures 2 and 3).

In 2013, Nature published 2 studies showing that circRNAs were newly found members of competing endogenous RNAs that could act as sponges of miRNA. CircRNAs can protect miRNA-targeted gene expression from degradation by interacting with miRNAs.^10,11^ Considering the function of circRNA, we speculated that *cir-ITCH* may act as a miRNA sponge to regulate miRNA expression. In our study, the qRT-PCR results showed that *cir-ITCH* could reduce the expression of miR-7, while the exact mechanism of the regulation remained to be investigated. Our research determined that *cir-ITCH* influenced the growth and metastasis of OS cells through miR-7 (Figure 4). We also confirmed the hypothesis with the rescue experiments in our research.

As research has shown that miR-7 can regulate drug resistance and progression in several cancers through the EGFR pathway,^15-18^ we further investigated the role of miR-7 and EGFR in OS. We found that *cir-ITCH* increased the EGFR protein level, which was consistent with the report that EGFR is a target gene of miR-7, and silencing *cir-ITCH* decreased the expression of EGFR in OS cells. EGFR is the receptor of EGF and is important for various cell signaling pathways. EGFR belongs to the ErbB receptor family, which includes ErbB-1, ErbB-2, ErbB-3, and ErbB-4. EGFR is also known as ErbB1 and HER1, and its mutation or overexpression generally triggers tumor development.^16,17^ Our results showed that miR-7 could decrease the protein level of EGFR accompanied by the reduction in EGFR phosphorylation (Figure 5). Inhibition of the EGFR pathway blocked the *cir-ITCH*-induced metastasis of OS cells (Figure 6). Thus, the rescue experiments verified that *cir-ITCH* promoted OS proliferation and metastasis through the *cir-ITCH*/miR-7/EGFR axis.

In future research work, we will further analyze the expression of *cir-ITCH* in patient tissues and its relationship with survival. We will also verify the relationship between this signaling pathway and metastasis in patient specimens and animal models.

As a rising star, circRNAs may play critical roles in diseases, especially in various cancers. Our research showed that *cir-ITCH* was abnormally highly expressed in OS. Furthermore, the *cir-ITCH*/miR-7/EGFR signaling pathway mediated the growth and metastasis of OS cells, which provided theoretical insight into the roles of circRNAs in the progression of OS. Thus, *cir-ITCH* may serve as a new biomarker and therapeutic target for OS.

## Abbreviations

CCK-8: Cell Counting Kit-8
circRNA: circular RNA
DMEM: Dulbecco’s modified Eagle’s medium
FBS: fetal bovine serum
miRNA: microRNA
ncRNA: noncoding RNA
OS: osteosarcoma
PCR: polymerase chain reaction
qRT-PCT: quantitative real-time polymerase chain reaction
